# Supplementary material for: Chronic RNA G-quadruplex accumulation in aging and Alzheimer’s disease
Source: eLife. 2025 Feb 24;14:RP105446. doi: 10.7554/eLife.105446 (PMC11850002; doi:10.7554/eLife.105446)
Supplement: Supplementary file 1. [file elife-105446-supp1.docx]

| **Antibody** | **Host** | **Source** | **Dilution** | **Target** |
| --- | --- | --- | --- | --- |
| BG4 | E.Coli monoclonal | Sigma-Aldrich MABE917 | 1:250 | G-quadruplex structure |
| Anti-flag M2 | Mouse monoclonal | Sigma-Aldrich F1804 | 1:500 | Flag-tag on BG4 |
| NeuN | Rabbit monoclonal | Cell Signaling 12943S | 1:1000 | Neurons |
| Phospho-tau S396 | Rabbit polyclonal | Invitrogen 44-752G | 1:500 | Tau phosphorylated at Ser396 |
| Phospho-tau T231 | Rabbit polyclonal | Invitrogen 44-746G | 1:500 | Tau phosphorylated at T231 |
| SOX10 | Rabbit monoclonal | Abcam ab155279 | 1:500 | Oligodendrocytes |
| GFAP | Rabbit polyclonal | Abcam EPR1034Y | 1:500 | Astrocytes |
| IBA1 | Rabbit polyclonal | WAKO 013-27691 | 1:1000 | Microglia |
| rRNA | Mouse monoclonal | Abcam ab17119 | 1:250/1:500 | Ribosomal RNA |
