# Supplementary material for: Chronic RNA G-quadruplex accumulation in aging and Alzheimer’s disease
Source: eLife. 2025 Feb 24;14:RP105446. doi: 10.7554/eLife.105446 (PMC11850002; doi:10.7554/eLife.105446)
Supplement: Supplementary file 2. — Highlighted column is the quantification included in the main text. All three R values are highly significant. [file elife-105446-supp2.docx]

|  |  | **Individual 1** | **Individual 2** | **Individual 3** |
| --- | --- | --- | --- | --- |
| **Age** | **Braak** | **Not Blinded** | **Blinded 1** | **Blinded 2** |
| 80 | 1.5 | 0.76 | 0.726 | 0.847 |
| 67 | 6 | 3.633 | 2.427 | 3.138 |
| 79 | 5 | 2.486 | 2.267 | 1.755 |
| 91 | 6 | 2.429 | 1.953 | 2.095 |
| 87 | 6 | 3.313 | 2.806 | 2.4 |
| 60 | 5.5 | 2.322 | 1.841 | 2.17 |
| 85 | 0 | 0.62 | 0.594 | 0.637 |
| 84 | 6 | 2.516 | 1.291 | 1.717 |
| 63 | 6 | 1.382 | 1.196 | 1.173 |
| 90 | 4.5 | 1.682 | 1.035 | 1.063 |
| 63 | 3.5 | 1.031 | 0.855 | 0.984 |
| 80 | 5 | 1.286 | 1.278 | 1.12 |
| 55 | 5 | 0.961 | 0.649 | 0.214 |
| 30 | 0 | 0.065 | 0.061 | 0.079 |
| 56 | 1.5 | 0.258 | 0.271 | 0.29 |
| 53 | 1.5 | 0.392 | 0.406 | 0.336 |
| 74 | 3 | 0.409 | 0.375 | 0.367 |
| 72 | 0 | 1.612 | 1.3 | 1.4 |
|  |  |  |  |  |
| **R value Age:** |  | 0.48 | 0.49 | 0.45 |
| **R value Braak:** |  | 0.74 | 0.7 | 0.65 |
